# LncRNA LINC01207 Could Positively Regulate the Development of Colorectal Cancer

**DOI:** 10.1155/2023/7671917

**Published:** 2023-02-24

**Authors:** Gaowu Hu, Wenquan Chen, Wei Peng, Yongqing Cao

**Affiliations:** ^1^Department of Anorectal Medicine, Shanghai Traditional Chinese and Western Medicine Integrated Hospital Affiliated to Shanghai University of Traditional Chinese Medicine, Floor 9, Building 5, No. 303, Changyang Road, Hongkou District, Shanghai, China; ^2^Department of Anorectal Medicine, Longhua Hospital Affiliated to Shanghai University of Traditional Chinese Medicine, 725 Wanping South Road, Shanghai, China

## Abstract

**Background:**

LINC01207 expression is associated with colorectal cancer progression. However, the exact role of LINC01207 in colorectal cancer (CRC) is not clear, and further exploration is needed.

**Methods:**

Gene expression data of the GSE34053 database were used to explore the differential expressed genes (DEGs) between colon cancer cells and normal cells. The gene expression profiling interactive analysis (GEPIA) was used to determine the differential expression of LINC01207 between CRC and normal tissues and the association between the expression of LINC01207 and survival in patients with CRC. The Kyoto Encyclopedia of Genes and Genomes (KEGG) and Gene Ontology (GO) analysis were performed to obtain the biological processes and pathways associated with DEGs and LINC01207 coexpressed genes in CRC. The qRT-PCR was used to determine the LINC01207 level in CRC cell lines and tissue samples. CCK-8 assay was employed to measure cell viability and Transwell assay to assess cell invasion and migration.

**Results:**

In this study, a total of 954 DEGs were identified, including 282 upregulated and 672 downregulated genes. LINC01207 was significantly upregulated in CRC samples with a poor prognosis. LINC01207 was also associated with pathways such as ECM-receptor interaction, O-glycan processing, and TNF signaling pathway in CRC. Knockdown of LINC01207 inhibited the migration, invasion, and proliferation of CRC cells.

**Conclusion:**

LINC01207 might act as an oncogene and promote the progression of CRC. Our study suggested that LINC01207 had the potential to be a novel biomarker for CRC detection and a therapeutic target for CRC treatment.

## 1. Introduction

Colorectal cancer (CRC) is one of the most common malignant tumors worldwide [1]. Approximately 900,000 people die from CRC each year, and the incidence is higher in the more economic regions [2]. In China, there are approximately 521,000 new cases and 248,000 deaths each year. The incidence rate ranks third among malignant tumors, and the mortality rate ranks fifth [3]. With the improvement of medical technology, some patients can be cured by surgery, adjuvant radiotherapy, and chemotherapy [4]. The 5-year survival rate of early-stage patients is about 80%–90%, but the survival rate is only 10% for advanced-stage patients [5]. To improve the overall survival rate of patients, early diagnosis and treatment are necessary.

As a special class of RNA molecule, long noncoding RNA (lncRNA) does not encode proteins. The length of lncRNA is more than 200 nucleotides [6]. Previous study indicated that abnormal expression of lncRNA was associated with human disease occurrence, including cancer, cardiovascular diseases, and degenerative neurological diseases [7]. It has been reported that the dysregulation of lncRNA expression can regulate various types of cancer progression, such as prostate cancer, bladder cancer, breast cancer, lung cancer, gastric cancer, and colorectal cancer. lncRNAs can lead to tumor metastasis, promote tumorigenesis, and increase chromosomal instability [8]. Therefore, it is necessary to further identify the function of lncRNAs in CRC.

Long noncoding RNA 1207 (LINC01207), located at 4q32, contains three exons and two introns. LINC01207 regulates gene transcription and protein translation [9]. LINC01207 is positively regulated in lung cancer [10] and pancreatic cancer [9], and its downregulation inhibits tumor growth and promotes apoptosis. LINC01207 could predict poor prognosis and inhibit cell metastasis by regulating the GSK-3 *β*/*β*-catenin signaling pathway in malignant glioma [11]. According to the data in the TCGA database [12], LINC01207 is associated with the prognosis of patients with CRC, indicating that LINC01207 may be an independent biomarker for CRC. Nevertheless, the present research on LINC01207's role in CRC is still limited, and the molecular mechanism remains unclear.

This study aimed to explore the biological effects and mechanisms of LINC01207 on CRC cell growth and invasion through bioinformatics and experimental analysis. LINC01207 expression was specifically increased in CRC tissues and cell lines. This may suggest LINC01207 as a new factor in CRC detection as a biomarker or therapeutic target.

## 2. Methods and Materials

### 2.1. Microarray Data

The GSE34053 mRNA expression data were obtained from the Gene Expression Omnibus (GEO) [13] database in the National Center of Biotechnology Information (NCBI) (https://www.ncbi.nlm.nih.gov/geo). GPL570 [HG-U133_Plus_2] Affymetrix Human Genome U133 Plus 2.0 Array was used in this study. Both the carcinoma cells and carcinoma-associated fibroblasts (CAF) from the same patient tumor were isolated and separately cultured. The CD133-positive colorectal cancer cells were set as the experimental group. CAF samples were used for the control group.

### 2.2. PPI Network Construction and Analysis

The Retrieval of Interacting Genes (STRING; string-db.org) database was used to perform the construction of the protein-protein interaction (PPI) network between DEGs. The confidence of threshold value for PPIs was set as (combined score) >0.7.

### 2.3. GEPIA Database

As a public online tool, gene expression profiling interactive analysis (GEPIA; https://gepia.cancer-pku.cn/) provides customizable functionalities based on data from The Cancer Genome Atlas (TCGA) and the Genotype-Tissue Expression project (GTEx). In the present study, through GEPIA, differential expression of LINC01207 graphically between colon cancer and normal tissues and the association between the expression of LINC01207 and survival in patients with CRC were determined.

### 2.4. GO and KEGG Pathway Enrichment Analyses

The Kyoto Encyclopedia of Genes and Genomes (KEGG) and Gene Ontology (GO) analyses both for DEGs and LINC01207 coexpressed genes were performed by The Database for Annotation, Visualization, and Integrated Discovery (DAVID, https://David.ncifcrf.gov/tools.jsp). The number of enriched genes >2 and *P* < 0.05 were set as cut-off criteria.

### 2.5. Clinical Tissue Samples

A total of 30 pairs of tumor tissue and adjacent normal tissue samples were collected from the Longhua Hospital Affiliated to Shanghai University of Traditional Chinese Medicine and stored in liquid nitrogen for subsequent experiments. This study was approved by the Ethics Committee of Longhua Hospital Affiliated to Shanghai University of Traditional Chinese Medicine, and all patients signed the informed consent.

### 2.6. Cell Lines and Cell Culture

The CRC cell lines (HRT-18, HCT-15, SW480, HCT-116, and RKO) and colorectal cell line FHC were obtained from the American Type Culture Bank (ATCC, Manassas, USA). Cells were maintained in RPMI-1640 (Gibco BRL, Karlsruhe, Germany) containing 10% fetal bovine serum (Gibco FBS, USA), 100 IU/mL penicillin, and 100 *μ*g/mL streptomycin (Baishitong, Chongqing, China).

### 2.7. Cell Transfection Assay

The si-LINC01207 #1, si-LINC01207 #2, and negative control (NC) were synthesized from GenePharma (Shanghai, China). Cells seeded in 6-well plates. The transfection was performed using Lipofectamine 2000 reagent (Invitrogen, USA) according to the manufacturer's instructions.

### 2.8. Cell Proliferation Assay (CCK-8)

The cell proliferation was assessed using a CCK-8 kit (Takara, Dalian, China). After 24 hours of transfection, HCT-116 and RKO (1 × 10^3^ cells per well) cells were seeded into 96-well plates. 10 *μ*l of CCK-8 reagent was added to each well at 0, 24, 48, and 72 hours, respectively. After incubation at 37°C for 1.5 h, the OD value of each well at 450 nm was measured with a microplate reader (BioRad, USA).

### 2.9. Quantitative Reverse Transcription-Polymerase Chain Reaction (qRT-PCR)

The total RNA from cells and tissues was isolated by Trizol reagent (Invitrogen). The cDNA was synthesized by a reverse transcription kit (Byotime, China). The qRT-PCR was performed using the SYBR Green Master Mix (Biosharp, China) on the ABI 7500 qPCR system (ABI, USA) with GAPDH as the internal reference gene. The primer mix was ordered from Kumei (Kumei, China). The reaction conditions were as follows: predenaturation at 95°C for 3 minutes, denaturation at 95°C for 2 hours, annealing at 60°C for 20 seconds, and extension at 72°C for 1 minute, a total of 40 steps. The relative expression was determined by 2^−∆∆Ct^ method.

### 2.10. Migration and Invasion Assays

The cell migration and invasion were determined by the Transwell method. Cells were plated into a 24-well plate with a serum-free medium at a density of 8 × 10^4^ cells per well. Subsequently, cells were washed and fixed in anhydrous methanol for 10 minutes and stained with DAPI for 10 minutes. Five different fields were randomly selected for observation.

### 2.11. Statistical Analysis

The statistical analysis was performed by using SPSS 18.0 software (SPSS Corporation, Chicago, Illinois, USA). The differences between two groups were determined by paired Student's *t*-test, and one-way analysis of variance (ANOVA) was used to determine differences between multiple groups. *P* < 0.05 indicated statistical significance.

## 3. Results

### 3.1. Analysis of DEGs

**Figure 1:**
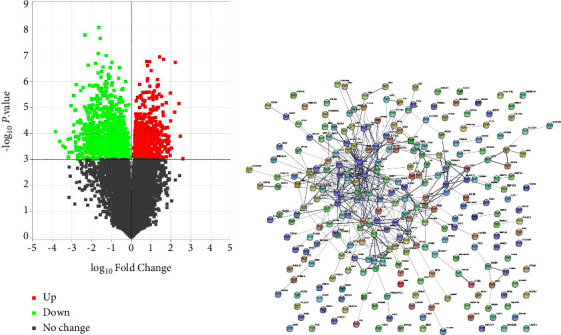
Analysis of DEGs between patients with colon cancer and controls. (a) Volcano plots of the aberrantly expressed genes between CD133-positive colorectal cancer cells and CAF according to GEO dataset GSE34053. Red dots represent upregulated genes defined as lgFC>1.0 and *P* < 0.001. Green dots indicate downregulated genes based on lgFC<−1.0 and *P* < 0.001. Gray dots represent mRNA expression with |lgFC|<1.0 and *P* > 0.001. FC, fold change. (b) Protein-protein interaction network analysis of DEGs.

To determine the DEGs between colon cancer cells and controls, the publicly available microarray dataset GSE34053 was obtained from the GEO database and the Limma package was analyzed. A total of 954 DEGs with the threshold of *P* < 0.001 and |Fold Change| >10 were identified, including 282 upregulated and 672 downregulated DEGs (Figure 1(a)). LINC01207 was identified as one of the upregulated genes. Subsequently, the PPI network was generated by using the STRING database. As shown in Figure 1(b), 271 nodes and 453 interaction pairs were determined (Figure 1(b)).

### 3.2. Association between High Expression of LINC01207 and Poor Prognosis

**Figure 2:**
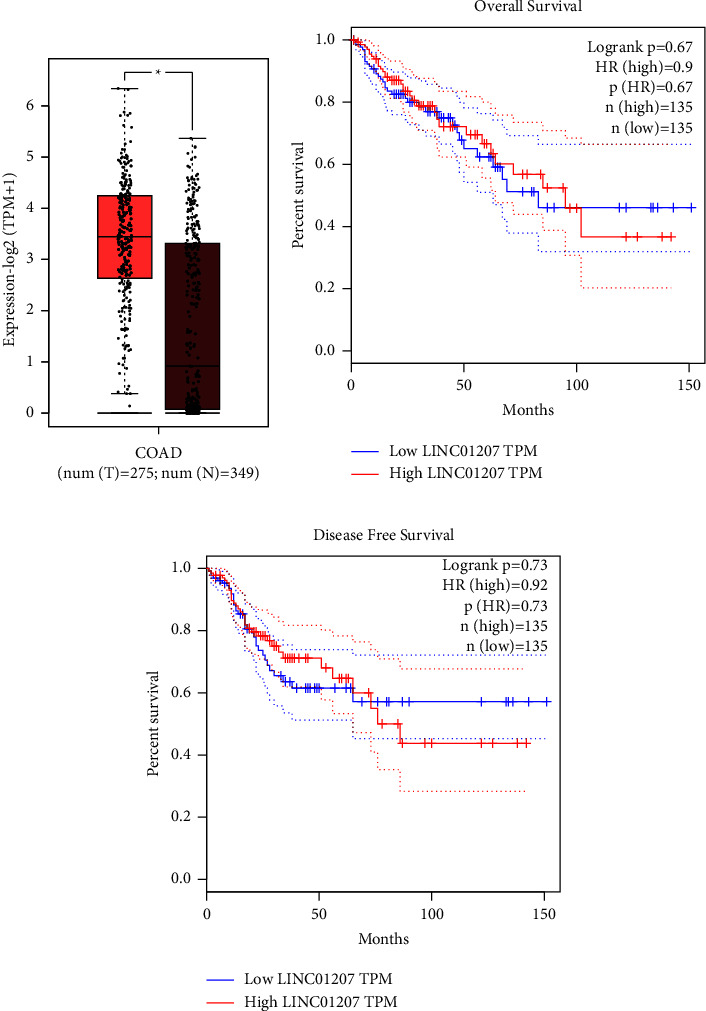
Association between high expression of LINC01207 and poor prognosis. (a) LINC01207 expression was significantly regulated positively in 275 CRC tissues in comparison with 349 normal colon tissues according to the GEPIA dataset. *P* < 0.05. (b) Association between LINC01207 expression level and overall survival of patients with CRC according to the GEPIA dataset. (c) Association between LINC01207 expression level and disease-free survival of patients with CRC based on the GEPIA dataset.

The molecular mechanism and prognostic value of LINC01207 in CRC were further investigated. As shown in Figure 2(a), LINC0120 was significantly upregulated in CRC tumor tissues (*n* = 275) compared with adjacent normal tissues (*n* = 349) based on the publicly available GEPIA dataset. To further evaluate the role of LINC01207 in prognosis, 270 patients with CRC from GEPIA were analyzed. As shown in Figures 2(b) and 2(c), both the overall survival and disease-free survival curve revealed that high LINC01207 expression was associated with an increased risk of mortality in patients with CRC compared to those with low LINC01207 expression. These results indicated that LINC01207 expression may serve as a prognostic biomarker in CRC.

### 3.3. Biological Processes and Pathway Enrichment Analyses

**Figure 3:**
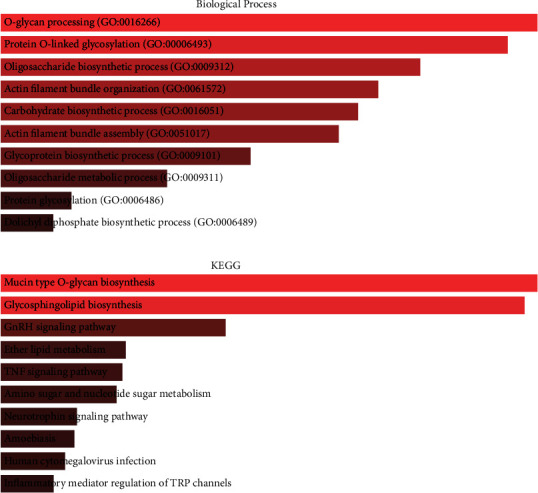
Enrichment analysis of differentially expressed genes. (a) Top 10 obviously enriched biological processes by GO analysis. (b) Top 10 enriched pathways by KEGG analysis.

**Figure 4:**
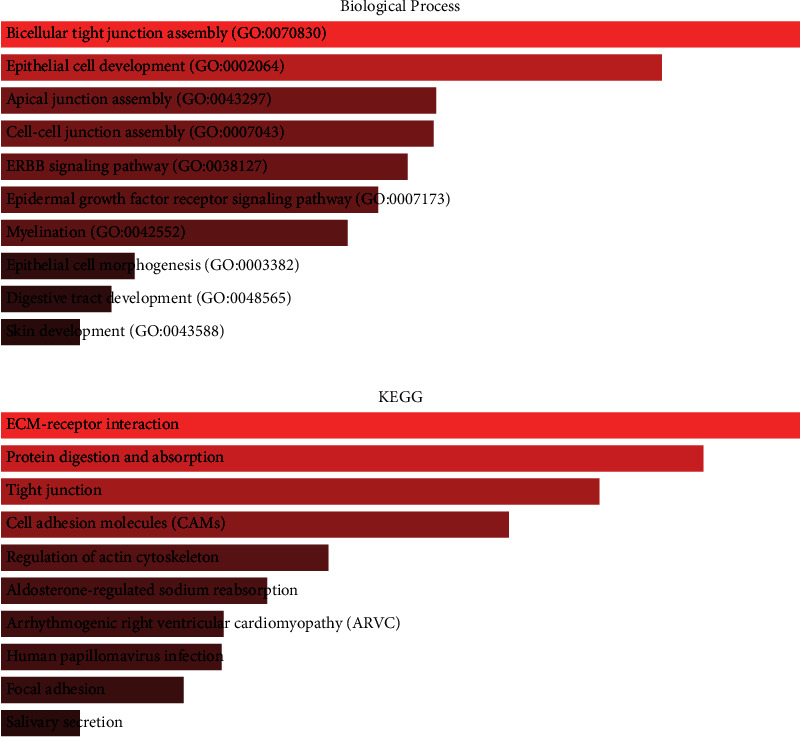
Enrichment analysis of LINC01207 coexpressed genes. (a) Top 10 obviously enriched biological processes by GO analysis. (b) Top 10 significantly enriched pathways by KEGG analysis.

To evaluate the molecular mechanisms in CRC initiation and progression, the GO and KEGG enrichment analyses for DEGs and LINC01207 coexpressed genes were performed. The results showed that the DEGs were significantly abundant in 193 GO biological processes and 13 KEGG pathways including epithelial cell development, ERBB signaling pathway, epidermal growth factor receptor signaling pathway, epithelial cell morphogenesis, ECM-receptor interaction, Cell adhesion molecules (CAMs), Human papillomavirus infection and Focal adhesion (Figures 3(a) and 3(b)). The coexpressed genes were abundant in 190 biological processes 26 KEGG pathways, such as O-glycan processing, protein O-linked glycosylation and oligosaccharide biosynthetic process, Mucin type O-glycan biosynthesis, Glycosphingolipid biosynthesis, GnRH signaling pathway, Ether lipid metabolism and TNF signaling pathway (Figures 4(a) and 4(b)).

### 3.4. The LINC01207 Level in CRC Samples and Cell Lines

**Figure 5:**
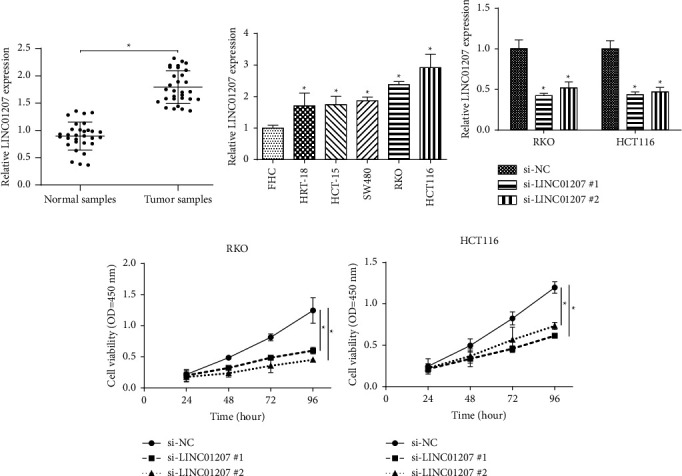
Increased expression of LINC01207 in CRC samples and cell lines. (a) Relative LINC01207 expression in CRC tumors and normal tissues. (b) Relative LINC01207 expression in CRC cell lines and normal cells. (c) LINC01207 was knocked down by si-LINC01207 #1 and si-LINC01207 #2 in HCT-116 and RKO cells. (d) The effect of LINC01207 knockdown on cell growth in RKO cells. (e) The effect of LINC01207 knockdown on cell growth in HCT-116 cells, ^*∗*^*P* < 0.05.

To explore the role of LINC01207 in CRC occurrence and development, the LINC01207 expression levels in CRC samples and cell lines were determined. As shown in Figure 5(a), the LINC01207 level in tumor tissues (*n* = 30) was significantly higher than that in normal samples (*n* = 30). Furthermore, LINC01207 levels in FHC, HRT-18, HCT-15, SW480, RKO, and HCT-116 cells were determined. The results indicated that LINC01207 was highly expressed in CRC cell lines (Figure 5(b)). These results suggested that LINC01207 may participate in CRC occurrence and development.

### 3.5. Silencing LINC01207 Inhibited CRC Cell Proliferation, Migration, and Invasion

**Figure 6:**
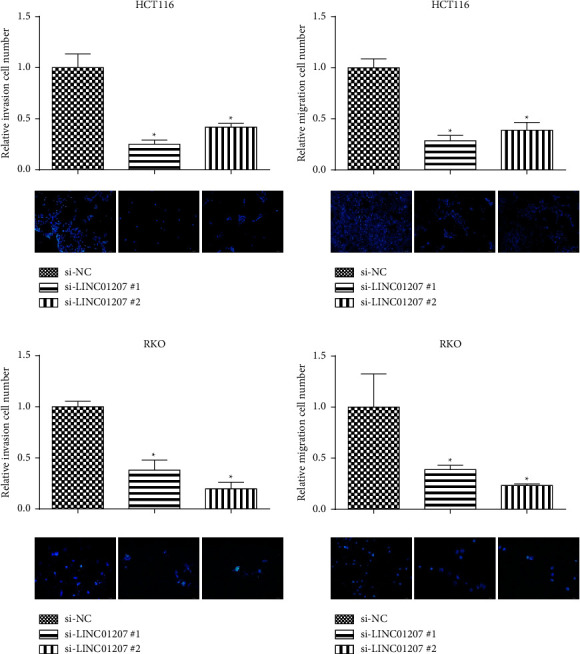
Silencing LINC01207 inhibited CRC cell migration and invasion. (a) Relative invasion cell number in HCT-116 cells transfected with si-LINC01207 #1 and si-LINC01207 #2. (b) Relative migration cell number in HCT-116 cells transfected with si-LINC01207 #1 and si-LINC01207 #2. (c) Relative invasion cell number in RKO cells transfected with si-LINC01207 #1 and si-LINC01207 #2. (d) Relative migration cell number in RKO cells transfected with si-LINC01207 #1 and si-LINC01207 #2, ^*∗*^*P* < 0.05.

To further determine the biological function of LINC01207 in CRC, expression of LINC01207 was inhibited by si-LINC01207 #1 and si-LINC01207 #2 in HCT-116 and RKO cells (Figure 5(c)). The CCK-8 analysis demonstrated that the knockdown of LINC01207 significantly inhibited cell proliferation of RKO (Figure 5(d)) and HCT-116 cells (Figure 5(e)). Additionally, Transwell migration and invasion assays indicated that LINC01207 knockdown also inhibited CRC cell migration and invasion (Figures 6(a)–6(d)). These results demonstrated LINC01207 was critical for CRC cell progression.

## 4. Discussion

CRC is a malignant tumor that occurs in the epithelium of the large intestine, most commonly in elderly patients [14]. Therefore, the disease is difficult to diagnose but easy to distant metastasis. The prognosis for patients with recurrent or metastatic disease is not ideal. For advanced colorectal cancer patients with liver or lung metastasis, the five-year survival rate is only about 5%–10% [15]. Therefore, it is very important to explore the molecular biological mechanism of CRC.

Bioinformatics analysis can identify candidate genes and help understand the genetic basis of diseases [16]. In the present study, mRNA expression data of GSE34053 obtained from GEO was analyzed by bioinformatics analysis. A total of 954 DEGs were screened, including 282 upregulated and 672 downregulated genes. Additionally, the PPI network of 282 upregulated DEGs was constructed to determine the close association of these genes in CRC. The LINC01207 was obviously upregulated in CRC and closed related to prognosis as determined by the online database GEPIA. The patients with high LINC01207 expression had shorter survival time. Therefore, LINC01207 has the potential to be a novel and valuable treatment and prognosis target in CRC.

Furthermore, GO and KEGG enrichment analyses both for DEGs and LINC01207 coexpressed genes were performed to evaluate the molecular mechanisms in CRC initiation and progression. The results revealed multiple biological processes and pathways associated with DEGs and LINC01207 coexpressed genes in CRC, such as epidermal growth factor receptor signaling pathway, ECM-receptor interaction, O-glycan processing, and TNF signaling pathway. These pathways were also reported to be critical for various cancers. For example, the ECM-receptor interaction signal pathway possibly participates in breast cancer development through transcriptome profiling [17]. O-glycan truncation in gastric cancer can enhance cancer-related functions of CD44 [18]. O-glycan-altered extracellular vesicles can act as a specific serum marker in pancreatic cancer [19, 20].

LINC01207 has been shown to be involved in various signaling pathways to regulate cancer development. In this study, the expression of LINC01207 in CRC clinical tissues and cells was high. CCK-8 and Transwell assays confirmed that LINC01207 could promote CRC cell proliferation, migration, and invasion. In summary, this study improves our understanding of the role of LINC01207 in CRC.

## 5. Conclusion

In conclusion, the bioinformatic analysis demonstrated that lncRNA LINC01207 may act as an oncogene that is highly expressed in CRC samples and associated with pathways such as ECM-receptor interaction, O-glycan processing, TNF signaling pathway in tumor growth, and metastasis. Moreover, our data demonstrated that LINC01207 can promote CRC cell migration, proliferation, and invasion. These findings suggested that LINC01207 had the potential to be a novel biomarker and target for CRC diagnosis and treatment.

## Data Availability

Data are available from the corresponding author upon request.
